# Multidimensional Assessment of Shoulder Biomechanics: Towards an Integrated Functional Interpretation

**DOI:** 10.3390/s26217080

**Published:** 2026-11-09

**Authors:** Umile Giuseppe Longo, Martina Sassi, Letizia Mancini, Matteo Di Benedetto, Davide Zolin, Pierfrancesco Crucitti, Michele Mercurio, Rocco Papalia, Emiliano Schena, Alessandro de Sire, Arianna Carnevale

**Affiliations:** 1 Fondazione Policlinico Universitario Campus Bio-Medico, Via Alvaro del Portillo, 200, 00128 Rome, Italy; g.longo@unicampus.it (U.G.L.); martina.sassi@policlinicocampus.it (M.S.); l.mancini@policlinicocampus.it (L.M.); dibenedettomatteo02@gmail.com (M.D.B.); zolindavide@gmail.com (D.Z.); pierfrancesco.crucitti@gmail.com (P.C.); r.papalia@policlinicocampus.it (R.P.); e.schena@unicampus.it (E.S.); arianna.carnevale@policlinicocampus.it (A.C.); 2 Research Unit of Orthopaedic and Trauma Surgery, Department of Medicine and Surgery, Università Campus Bio-Medico di Roma, Via Alvaro del Portillo, 21, 00128 Rome, Italy; 3 Department of Orthopedic and Trauma Surgery, “Magna Graecia” University, “Renato Dulbecco” University Hospital, 88100 Catanzaro, Italy; michele.mercurio@unicz.it; 4 Research Center on Musculoskeletal Health, MusculoSkeletalHealth@UMG, “Magna Graecia” University, 88100 Catanzaro, Italy; 5 Research Unit of Measurement and Biomedical Instrumentation, Department of Engineering, Università Campus Bio-Medico di Roma, 200, 00128 Rome, Italy; 6 Department of Life Sciences, Health, and Health Professions, Link Campus University, 00165 Rome, Italy

**Keywords:** shoulder, biomechanics, kinematics, rehabilitation, orthopedics, analysis

## Abstract

The assessment of shoulder function remains challenging because static imaging and isolated clinical measures may not fully capture the behaviour of the shoulder complex during dynamic tasks. Instrumented biomechanical analysis provides quantitative information on shoulder kinematics, intersegmental coordination, kinetics, and neuromuscular activation; however, its clinical interpretation remains limited by task dependency, methodological variability, and the complexity of integrating multiple biomechanical outputs. This narrative review synthesizes foundational and contemporary literature on shoulder biomechanics during dynamic upper-limb tasks. Scapular-plane arm elevation is proposed as a pragmatic reference task, with shoulder motion represented through a normalized elevation cycle to provide a common temporal framework for interpretation. Biomechanical outputs are organized into complementary domains encompassing scapulothoracic and glenohumeral kinematics, intersegmental coordination, kinetics, and neuromuscular contribution. An integrated approach to the interpretation of shoulder biomechanics is presented, emphasizing the relationships among scapulothoracic and glenohumeral motion, their intersegmental coordination, and, when available, kinetic and neuromuscular information. Three recurrent functional phenotypes are described: altered scapular contribution, compensatory trunk–shoulder strategies and altered humeral axial rotation coupling. The value of shoulder biomechanical assessment lies in the integrated interpretation of movement patterns rather than in isolated variables. This multidimensional perspective provides a framework for translating biomechanical data into functionally meaningful information for clinics and research.

## 1. Introduction

The assessment of shoulder function remains a persistent challenge in clinical practice. Despite advances in imaging and clinical examination, static measures often fail to capture the behaviour of the shoulder complex during dynamic, functional tasks [1,2]. Pain, weakness, or instability may become apparent only during movements that require coordinated contributions from multiple joints and muscle groups, thereby limiting the explanatory value of isolated anatomical or strength-based assessments [3,4].

Instrumented motion analysis provides a means of quantifying shoulder function during active movement, offering objective measures of kinematics, kinetics, coordination, and neuromuscular activation [5,6,7]. However, the clinical utility of these data remains constrained by challenges in their interpretation [8]. Unlike lower-limb biomechanics, where gait analysis benefits from standardized tasks, well-defined movement cycles, and established interpretive conventions, shoulder biomechanics encompasses heterogeneous tasks, diverse modelling approaches, and inconsistent terminology [9]. Consequently, similar biomechanical outputs may be interpreted differently across studies and clinical settings, limiting their comparability and translational value.

The shoulder complex presents unique biomechanical characteristics that further contribute to this interpretive challenge. Upper-limb motion results from the coordinated contribution of the glenohumeral (GH), scapulothoracic (ST), acromioclavicular, and sternoclavicular articulations, enabling complex and multiplanar movements [10]. Scapular motion, in particular, plays a critical role in facilitating humeral elevation, orienting the glenoid relative to the humeral head, and providing a functional base for upper-limb movement. However, accurate in vivo quantification of scapular kinematics remains challenging, particularly because of soft-tissue artefact associated with skin-based measurement techniques [11]. These characteristics complicate direct comparison across tasks and individuals, and highlight the need for interpretive frameworks that extend beyond isolated joint angles.

The shoulder biomechanics literature reports a wide array of kinematic, kinetic, and neuromuscular variables, reflecting the multidimensional nature of shoulder function [3]. Although these measures are becoming increasingly accessible through laboratory-based and emerging wearable and markerless technologies, their routine clinical adoption remains limited by practical and methodological barriers [12]. Laboratory-based motion analysis typically requires specialized equipment, controlled environments, trained personnel, and time-consuming acquisition and data processing procedures, while wearable and markerless systems, although more portable, may be affected by sensor placement, calibration, drift, camera configuration, and task-dependent measurement accuracy [12,13,14,15]. Recent systematic reviews have reported variable reliability and validity across shoulder movements, with greater inconsistencies observed for complex and multidirectional tasks [16,17]. These limitations may hinder reproducibility across clinical settings and complicate the translation of biomechanical measurements into clinically meaningful information. Clinicians may therefore be presented with detailed reports containing multiple curves and metrics without clear guidance on which variables are most informative, how they should be interpreted in relation to one another, or how these findings should be integrated into clinical reasoning. In the absence of structured interpretive approaches, biomechanical data may therefore be underutilized or misinterpreted.

To inform the development of this interpretive framework, a structured literature search was conducted across three electronic databases (PubMed, Scopus, and Web of Science), covering publications from January 2010 to January 2026. The search focused on adult human studies investigating shoulder biomechanics during active upper-limb tasks using instrumented assessment methods, including kinematic, kinetic, and neuromuscular measures. Study selection was guided by relevance to biomechanical measurement and interpretation, with priority given to in vivo dynamic human data. Purely computational or simulation-only studies without experimental validation, as well as animal, cadaveric, pediatric, conference abstracts, editorials, expert opinions, and single-case reports, were excluded. Title and abstract screening were followed by full-text assessment by the reviewers, with disagreements resolved by consensus and, when necessary, by adjudication by a fourth reviewer. No formal risk-of-bias assessment or quantitative meta-analysis was performed; the synthesis was qualitative and interpretive, and the functional phenotypes described below represent descriptive interpretive patterns rather than statistically derived or clinically validated clusters. The complete database-specific search strategies are provided in Table 1.

The aim of this narrative review is to synthesize existing evidence on shoulder biomechanics and propose an integrated framework for the functional interpretation of commonly reported shoulder biomechanical outputs during dynamic upper-limb tasks. Rather than exhaustively reviewing the literature or establishing diagnostic thresholds, this review seeks to clarify the biomechanical significance of individual variables, their interrelationships during functional movement, and methodological considerations on their interpretation. The main contribution of this review is the organization of these multidimensional biomechanical findings into a structured interpretive framework, using scapular-plane arm elevation as a reference task and identifying three recurrent functional phenotypes as descriptive movement patterns rather than clinically validated classifications. This approach aims to support clinicians and researchers in translating instrumented motion analysis data into functionally meaningful insights about shoulder function [8].

## 2. The Shoulder Task Cycle

Shoulder biomechanics encompasses a wide range of movements that differ substantially in their kinematic, kinetic, and neuromuscular demands. Consequently, the interpretation of biomechanical outputs is inherently dependent on the task being performed. The absence of a shared reference task and temporal framework further complicates the comparison and integration of findings across individuals, clinical populations, and experimental settings. Individual biomechanical variables may therefore be difficult to interpret without considering their relationship with other biomechanical outputs and the specific demands of the task.

Beyond task dependency, shoulder motion is characterized by continuously changing contributions from the different components of the shoulder complex throughout movement [10]. During arm elevation, the relative contributions of GH and ST motion vary across the range of elevation rather than occurring as discrete sequential phases. Classic observations of shoulder motion highlighted this coordinated behaviour, together with considerable inter-individual variability, particularly during the early phase of elevation [18]. These characteristics reinforce the need to interpret biomechanical variables not only within the context of the task being performed, but also according to how they evolve throughout the movement.

### 2.1. Rationale for a Reference Task in Shoulder Biomechanics

A common functional reference can facilitate the interpretation and comparison of biomechanical data across studies, clinical populations, and measurement settings. An appropriate reference task should be reproducible, biomechanically informative, clinically recognizable, and sufficiently general to be applied across different shoulder conditions without being diagnosis-specific.

Among the upper-limb movements commonly used to assess shoulder function, scapular-plane arm elevation, or scaption, provides a practical reference across biomechanical research, clinical assessment, and rehabilitation settings [2,19]. Scaption consists of arm elevation in the scapular plane, typically oriented approximately 30–45° anterior to the frontal plane [20], and provides a controlled yet functionally relevant task for investigating ST and GH kinematics and their inter-segment coordination throughout the range of elevation.

However, scaption does not capture the full spectrum of shoulder function, and task-specific assessments remain necessary to investigate the distinct mechanical and coordinative demands of more complex functional or sport-specific movements.

### 2.2. Conceptual Definition of the Shoulder Elevation Cycle

**Figure 1:**
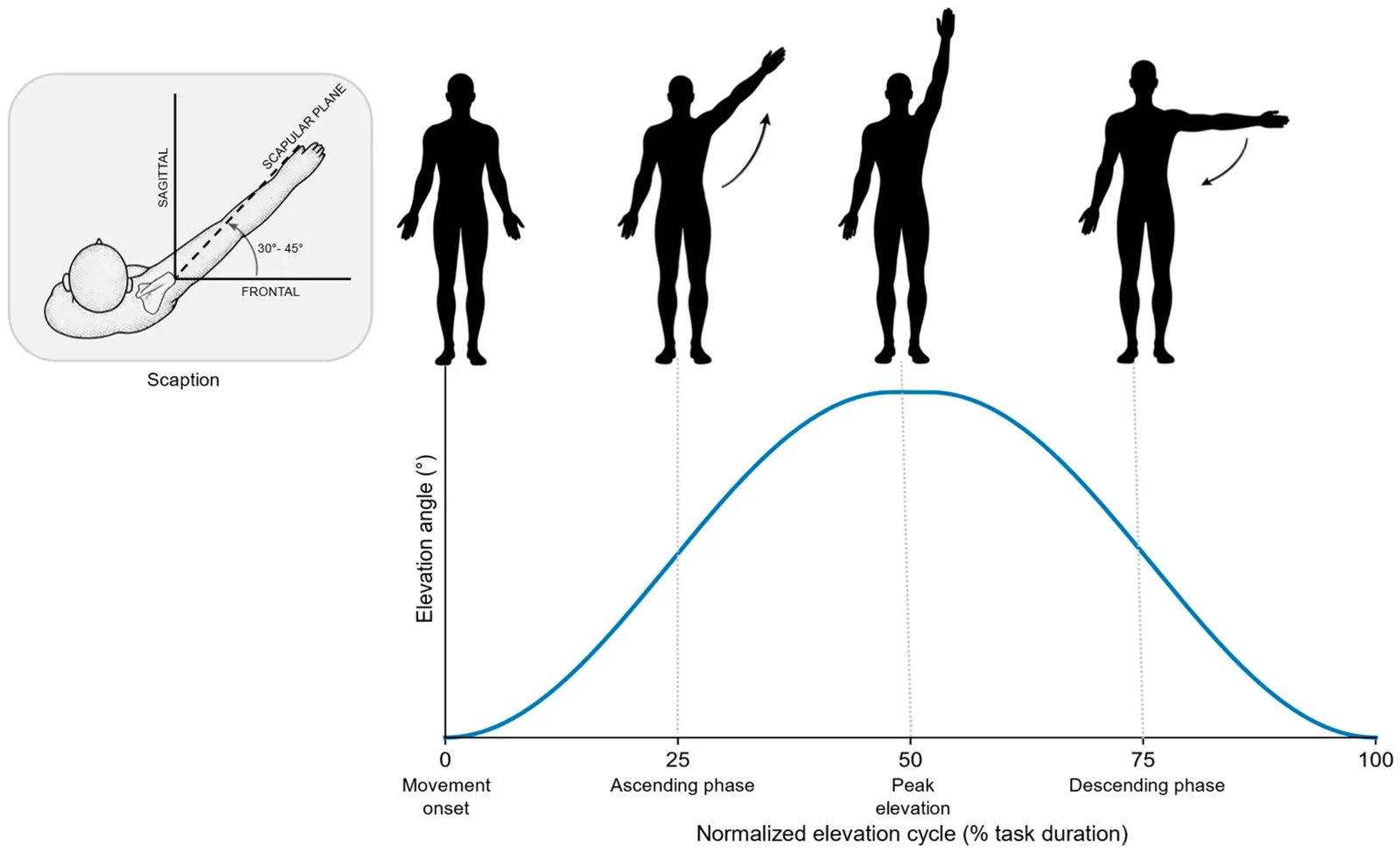
Conceptual normalized shoulder elevation cycle during scapular-plane arm elevation (scaption). Scaption is illustrated as arm elevation in a plane oriented approximately 30–45° anterior to the frontal plane. The cycle is divided into movement onset, ascending phase, peak elevation, and descending phase, providing a shared temporal reference for interpretation of shoulder kinematics, inter-segment coordination, kinetics, and neuromuscular timing across the task.

Considering scaption as a reference task, shoulder motion can be represented as a normalized elevation cycle that provides a common temporal framework for biomechanical and neuromuscular interpretation. Figure 1 provides a conceptual illustration of the shoulder elevation cycle and does not represent original experimental measurements or data-derived trajectories. Four key events and phases can be identified within this cycle. Movement onset corresponds to the initiation of humeral elevation from the resting position and marks the beginning of the ascending phase, during which the arm is progressively elevated in the scapular plane. Peak elevation corresponds to the maximum humeral elevation reached during the task and marks the transition to the descending phase, during which the arm is lowered toward the initial position.

Time-normalizing the elevation task to a percentage-based cycle facilitates comparison across individuals and trials by accounting for differences in movement duration while preserving the temporal progression of biomechanical variables.

## 3. Biomechanical Domains and Core Outputs

Shoulder motion analysis provides a range of outputs describing different aspects of movement, including segment and joint kinematics, intersegmental coordination, and, when available, kinetic and neuromuscular variables [8,10,21]. For interpretation, these outputs can be organized into complementary biomechanical domains that describe different but interrelated aspects of shoulder function. Their clinical relevance therefore depends not only on individual measures, but also on how they relate to one another within the movement being examined.

### 3.1. Scapulothoracic Kinematics

Scapulothoracic kinematics describe the motion of the scapula relative to the thorax and represent a key component of shoulder biomechanics [6,22]. During arm elevation, scapular motion contributes to the orientation of the glenoid relative to the humeral head and provides a continuously adapting base for humeral movement and muscle function [23,24,25].

Scapular orientation is commonly described by three rotational components: upward rotation, posterior tilt, and external rotation [6]. During arm elevation, the scapula typically exhibits increasing upward rotation, posterior tilt, and external rotation, although the magnitude and temporal evolution of these components vary across individuals and task conditions. These rotations occur concurrently and should therefore be considered as components of a coordinated three-dimensional movement rather than interpreted independently.

From an interpretive perspective, ST kinematics characterize how scapular orientation changes throughout arm elevation and provide insight into the contribution of scapular motion to the overall humeral elevation strategy. Interpretation should, however, account for the task performed and the measurement approach used, particularly because scapular kinematics are sensitive to tracking methods and soft-tissue artefact [11,26,27].

### 3.2. Humeral and Glenohumeral Kinematics

Humeral kinematics describe the orientation and motion of the arm relative to a defined reference frame, commonly the thorax, whereas glenohumeral kinematics describe humeral motion relative to the scapula. Core descriptors include humeral elevation, plane of elevation, and axial (internal–external) rotation [6]. Together, these variables characterize the three-dimensional orientation of the humerus throughout the movement.

Distinguishing humerothoracic (HT) from GH motion is essential when interpreting arm elevation. HT elevation reflects the combined contributions of GH and ST motion relative to the thorax, whereas global humeral elevation may additionally be influenced by compensatory trunk movements. GH kinematics, in contrast, specifically characterize humeral motion relative to the scapula. Interpretation should also account for the limitations of non-invasive measurement techniques, as small between-group differences in GH kinematics may approach or fall below measurement resolution [28].

According to the International Society of Biomechanics (ISB) recommendations, shoulder kinematics should be described using anatomically defined coordinate systems for the thorax, scapula, and humerus [6]. The distinction between HT and GH motion can be expressed through the corresponding rotation matrices. If $R_{TH}$ represents the orientation of the humerus relative to the thorax and $R_{TS}$ represents the orientation of the scapula relative to the thorax, GH orientation can be calculated a:

$$R_{SH}=R_{TS}^{-1}\;R_{TH}$$

assuming consistent rotation-matrix conventions. Therefore, accurate estimation of GH kinematics requires information about both humeral and scapular orientation, whereas humeral tracking alone cannot distinguish GH motion from the contribution of the scapula.

Axial rotation provides additional information on humeral orientation throughout elevation and on how rotational behaviour changes as elevation progresses [29]. Its interpretation is therefore particularly informative when considered in relation to humeral elevation, allowing axial rotation–elevation coupling to be characterized throughout the movement rather than relying solely on values measured at isolated time points.

### 3.3. Inter-Segment Coordination: Scapulohumeral Rhythm

Inter-segment coordination describes how the different components of the shoulder complex contribute to and interact during movement. Within this domain, scapulohumeral rhythm describes the relative contributions of scapular and humeral motion during arm elevation [30].

Scapulohumeral rhythm should be understood as a dynamic relationship rather than as a fixed ratio. Although historically expressed as a numerical relationship between humeral and scapular motion, their relative contributions change throughout arm elevation and vary across tasks, loading conditions, and individuals [30].

Task-specific studies have demonstrated different patterns of GH and ST contribution across upper-limb exercises [31], while inter-individual variability limits the applicability of population-average ratios to individual movement patterns [32]. Changes in scapulohumeral coordination may also influence muscle force distribution and joint loading, further supporting the functional relevance of this relationship [33].

From an interpretive perspective, scapulohumeral rhythm provides information on shoulder coordination that may not be captured by examining scapular or glenohumeral kinematics independently [4]. Similar overall arm elevation may be achieved through different relative contributions of glenohumeral and scapulothoracic motion. Accordingly, examining how these contributions evolve throughout the elevation task can help characterize the movement strategy used to achieve arm elevation.

### 3.4. Kinetics and External Loading

Kinetic variables describe the forces and moments associated with shoulder movement and provide information on the mechanical demands exerted during a given task. Kinetic analysis is less commonly incorporated into shoulder motion studies than kinematic analysis, reflecting the additional methodological requirements associated with external load quantification, segmental modelling, and inverse dynamics [10].

When available, kinetic outputs such as net joint moments and externally applied forces complement kinematic analysis by providing information on the mechanical demands associated with observed movement patterns. Similar kinematic profiles may occur under substantially different loading conditions, depending on task execution, external resistance, and the underlying mechanical context [23]. Kinetic interpretation should also consider the direction and phase of movement, as joint moments and mechanical demands may differ between the ascending and descending phases of arm elevation, particularly under external loading conditions. Model-based simulations further demonstrate that GH joint reaction forces during scapular-plane elevation are sensitive to prosthetic configuration and rotator cuff function [34].

Interpretation of kinetic outputs should therefore account for the loading conditions, modelling approach, and assumptions underlying their estimation [10]. Given their methodological sensitivity and more limited availability, kinetic variables are considered complementary to the kinematic characterization of shoulder movement.

### 3.5. Neuromuscular Contribution

Surface electromyography (sEMG) provides indirect information on muscle activation patterns associated with shoulder movement. Relevant outputs include the timing, amplitude, and coordination of muscle activation throughout the task [35]. Their interpretation requires consideration of methodological factors, particularly electrode placement and normalization procedures, which can substantially influence amplitude-based measures [35,36]. Common sEMG normalization approaches include normalization to maximum voluntary isometric contraction (MVIC) or to a reference value obtained during a specific movement task. These approaches provide different reference conditions and may influence comparisons of muscle activation amplitudes across individuals, tasks, and movement phases [35,36]. Therefore, the normalization procedure should be explicitly reported when interpreting neuromuscular data.

Neuromuscular data complement kinematic analysis by providing information on muscle activation strategies that cannot be inferred from movement trajectories alone. Similar kinematic patterns may be associated with different activation timing and amplitude across individuals, movement directions, and task demands [37]. Movement direction may also influence neuromuscular strategies. Comparisons between lifting and lowering tasks have demonstrated different patterns of multi-muscle activation despite comparable joint kinematics [38]. Neuromuscular demands may also vary across different phases of high-demand upper-extremity tasks, with peak muscle activation and joint loading occurring at specific phases of the movement [39]. Fatigue provides a further example of the complementary information offered by neuromuscular assessment. Under fatigue conditions, muscle activation demands may increase even when peak joint angles remain relatively unchanged, suggesting that similar kinematic outcomes may be maintained through different neuromuscular demands [40]. Fatigue-related adaptations may also vary across individuals, with different muscle groups contributing to load redistribution during the same task [41].

Shoulder muscle activation may also be influenced by proximal factors beyond local shoulder motion. Changes in lumbopelvic posture, for example, have been associated with altered activation of scapular muscles even in the absence of observable changes in shoulder position [42]. These findings reinforce the need to interpret electromyographic data within a whole-body context, particularly when assessing compensatory strategies or intervention effects.

### 3.6. Integrating Domains for Interpretation

Shoulder function is best characterized by considering ST and GH kinematics together with their intersegmental coordination within the context of the movement task [4].

**Figure 2:**
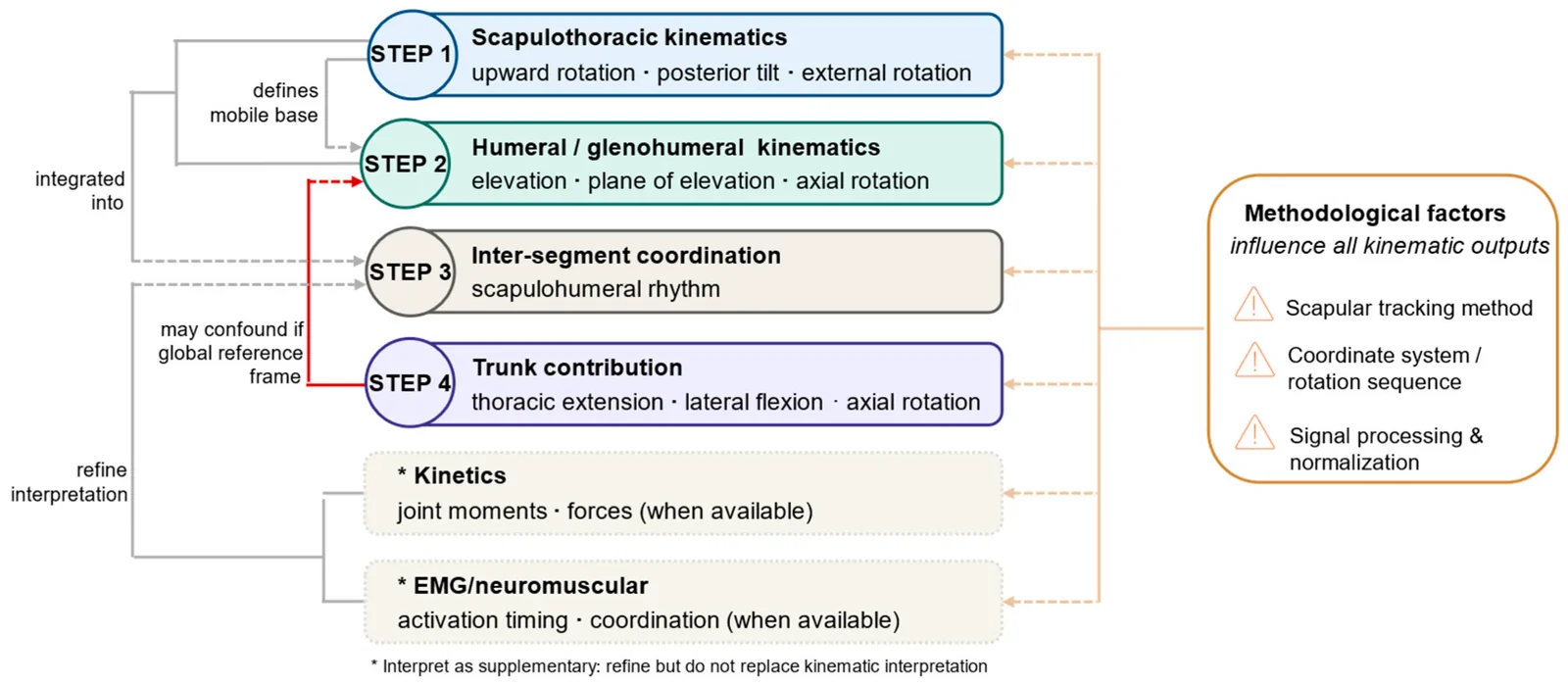
Biomechanical domains and interpretive dependencies across the shoulder elevation cycle. Scapulothoracic kinematics define the mobile base for humeral and glenohumeral motion, inter-segment coordination links scapular and humeral behaviour, and trunk contribution may confound the interpretation of apparent global humeral elevation when a global reference frame is used. Kinetic and neuromuscular outputs, when available, provide complementary information that refines but does not replace kinematic interpretation. Methodological factors, including scapular tracking, coordinate system definition, and signal processing choices, may influence reported biomechanical outputs. Grey dashed arrows represent conceptual interpretive relationships, red dashed arrows indicate potential confounding of apparent global humeral elevation due to trunk motion, and orange dashed arrows indicate methodological influences. Warning triangles identify potential sources of measurement variability. The connections are conceptual and do not imply experimentally established causal relationships.

When available, kinetic and neuromuscular data provide complementary information on the mechanical and muscular demands associated with the observed movement pattern [1,43]. Integrating these biomechanical domains provides the basis for interpreting different shoulder movement strategies and for identifying the recurrent functional phenotypes discussed in the following section. Figure 2 provides a conceptual schematic illustrating the integration of biomechanical domains and the methodological factors influencing their interpretation, rather than representing original experimental data.

## 4. Clinical Interpretation by Biomechanical Phenotype

For clinical interpretation, shoulder biomechanical outputs can be considered as part of broader functional phenotypes rather than as isolated variables or diagnosis-specific markers [4]. These phenotypes describe recurrent patterns of movement and coordination that may be observed across different shoulder conditions and functional demands [3]. Different conditions may present similar biomechanical features, while different movement strategies may occur within the same diagnostic category [44]. The phenotypes described below do not represent formally derived clusters or diagnostic classifications, but recurrent interpretive patterns that can be used to integrate kinematic and coordination findings with kinetic and neuromuscular information, when available. They should therefore be interpreted as task-dependent descriptions of shoulder movement strategies rather than as indicators of a specific pathology.

### 4.1. Altered Scapular Contribution Phenotype

A commonly reported biomechanical pattern involves alterations in the contribution of scapular motion during arm elevation [45,46]. This phenotype is characterized by changes in the timing and magnitude of ST motion relative to humeral elevation, rather than by isolated abnormalities in a single scapular variable [47].

Biomechanically, this pattern may manifest as changes in the magnitude or timing of scapular upward rotation and posterior tilt during arm elevation [1]. In some cases, these alterations are accompanied by increased reliance on humeral motion in the early phases of the task, followed by compensatory increases in scapular contribution at higher elevation angles. Importantly, such patterns may occur even when peak scapular angles fall within expected ranges, underscoring the importance of examining temporal behaviour across the elevation cycle [44]. Importantly, alterations in scapular contribution are not necessarily indicative of pathology and may also reflect task-dependent or adaptive movement strategies. Sport-specific investigations have shown that experienced athletes preserve scapular posterior tilt and limit unfavourable humeral positioning under high joint-loading conditions, thereby maintaining acromiohumeral clearance, whereas less experienced individuals exhibit reduced scapular control and greater compromise of subacromial space [48]. Similarly, experimental manipulation of rehabilitation tasks demonstrates that scapular contribution during elevation is modifiable, with different exercise selections eliciting distinct coordination strategies characterized by either ST-dominant or GH-dominant patterns of motion sharing [31].

From a biomechanical perspective, this phenotype reflects changes in how arm elevation is distributed across the components of the shoulder complex. ST motion itself results from coordinated motion at the sternoclavicular and acromioclavicular joints [49], emphasizing that altered scapular contribution should not be interpreted as the isolated behaviour of a single joint or muscle. Such patterns may be associated with pain, changes in neuromuscular function, or task-dependent adaptations [3,4].

From an interpretive perspective, recognizing this phenotype shifts attention away from isolated static scapular positioning toward dynamic coordination, informing rehabilitation strategies that target timing and integration of scapular motion rather than isolated strengthening. Clinical maneuvers intended to manually modify scapular position may alter symptoms without consistently restoring elevation strength or underlying movement mechanics, underscoring the distinction between short-term symptom modulation and functional normalization [50]. Such alterations are increasingly interpreted as modifiable movement strategies rather than fixed structural abnormalities, reinforcing the clinical relevance of dynamic scapular control over static positioning [51].

### 4.2. Compensatory Trunk–Shoulder Strategy Phenotype

A second biomechanical phenotype involves increased trunk contribution to the achievement of arm elevation [52]. In this pattern, apparent increases in functional range of motion (ROM) are achieved through coordinated thoracic extension, lateral flexion, or axial rotation rather than through proportional increases in GH or ST motion [52,53].

Biomechanically, this phenotype is characterized by preserved or even exaggerated humeral elevation curves when referenced to the global frame, coupled with altered inter-segment coordination between the trunk and upper limb. When scapular tracking is available, ST and GH contributions can be examined separately to characterize how movement is redistributed across the shoulder complex. In contrast, when only global humeral orientation is measured, apparent preservation of arm elevation may reflect compensatory trunk movement rather than preserved HT or GH function [52]. Compensatory trunk-shoulder strategies have been consistently documented across clinical populations performing functional reaching tasks. In neurologically impaired individuals, increased trunk flexion, shoulder girdle elevation, and distal joint involvement have been shown to substitute for reduced proximal shoulder and elbow contribution, enabling task completion at the cost of altered movement quality and modified inter-segment coordination [54,55,56]. Importantly, these compensatory patterns are quantifiable using both laboratory-based and wearable measurement systems, and their decomposition into component kinematic contributions has been shown to inform the identification of maladaptive versus task-enabling strategies, with direct implications for targeted rehabilitation planning, which may help inform rehabilitation planning when interpreted alongside clinical findings [54,56].

Clinically, compensatory trunk strategies can mask underlying shoulder limitations and lead to misinterpretation of apparent functional capacity. Without concurrent assessment of trunk motion, such compensations may be mistaken for preserved shoulder mechanics [52]. Identifying this phenotype is therefore critical when interpreting motion analysis data, as it highlights the need to distinguish true shoulder function from task-level adaptations. In practical terms, this recognition informs both clinical assessment and intervention planning, emphasizing the importance of addressing global movement strategies rather than focusing exclusively on local joint measures.

### 4.3. Altered Axial Rotation and Coupling Phenotype

A third interpretive phenotype involves alterations in humeral axial rotation and its coupling with elevation during arm motion [57]. This pattern is less readily identifiable through static measures but becomes apparent when examining the temporal relationship between elevation and axial rotation across the task cycle.

Biomechanically, this phenotype may present as reduced variability in axial rotation, delayed rotational adjustments during elevation, or atypical coupling between elevation and rotation phases [57]. These alterations may be associated with differences in how the shoulder coordinates joint motion and muscular demands during dynamic movement [5]. Recent sport- and task-specific studies have reported alterations in axial rotation timing and coupling in association with shoulder pain or high-demand tasks, suggesting that these measures may provide additional descriptive information beyond peak kinematics [58,59]. Similarly, experimental studies examining fatigue and chronic shoulder pain have reported changes in intersegmental coordination and variability even when peak angular values appear unremarkable, suggesting that coupling metrics may capture temporal features of movement that are not reflected by peak kinematic values alone [59]. Unlike overall range-of-motion deficits, such patterns may provide additional information about movement control that is not captured by peak kinematic values alone. Importantly, the mechanical implications of alterations in axial rotation coupling and scapulohumeral coordination remain incompletely established. Numerical analyses in surgical populations have suggested that altered shoulder rhythm may be associated with differences in estimated muscle and joint forces after reverse shoulder arthroplasty [33]. These findings support considering coordination measures alongside conventional kinematic variables, including when gross kinematic abnormalities are not apparent.

From a clinical standpoint, altered axial rotation coupling may provide additional descriptive information even when overt kinematic restrictions are not present. Its identification requires an integrative interpretation of humeral and scapular kinematics rather than reliance on peak values. Recognizing this phenotype may provide a more nuanced description of shoulder function by complementing conventional assessment of mobility and strength with information on dynamic control and coordination. Comparable alterations in axial rotation behaviour have also been reported in older adults with rotator cuff pathology during activities of daily living, where increased internal rotation and reduced elevation excursion have been interpreted as compensatory movement strategies despite preserved task completion [60].

**Figure 3:**
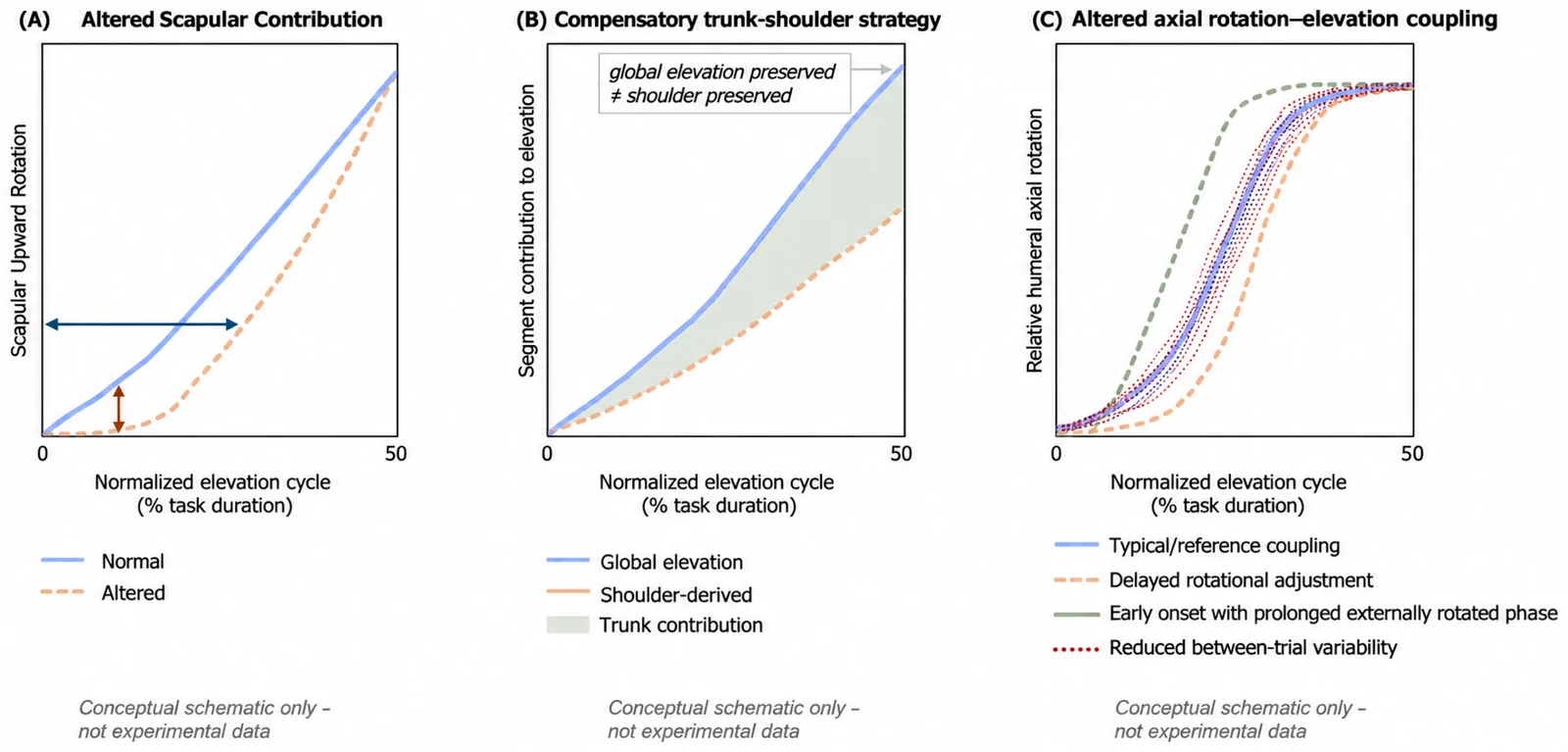
Conceptual schematic of three recurrent biomechanical phenotypes for clinical interpretation of shoulder motion. Panels depict the ascending half of the conceptual normalized elevation cycle from 0 to 50 percent, corresponding to the arm raising phase of the reference task, while the descending phase is omitted for clarity. (**A**) Altered scapular contribution phenotype. The orange dashed curve labelled Altered, compared with the solid blue curve labelled Normal, illustrates reduced early scapular upward rotation magnitude, indicated by the orange vertical double headed arrow, and delayed scapular contribution during elevation, indicated by the blue horizontal double headed arrow, with a later increase toward similar peak values. (**B**) Compensatory trunk shoulder strategy phenotype. The solid blue curve represents global elevation, the orange dashed curve represents the shoulder derived contribution, and the shaded area illustrates trunk contribution. Preserved global elevation may be achieved through increasing trunk contribution despite reduced shoulder derived contribution, potentially masking underlying shoulder limitation. (**C**) Altered axial rotation elevation coupling phenotype. Relative to the solid blue curve representing Typical or reference coupling, the orange dashed curve illustrates delayed rotational adjustment and the green dashed curve illustrates early onset with a prolonged externally rotated phase, despite relatively preserved peak rotational magnitude. The thin red dotted curves schematically illustrate the concept of reduced between trial variability as labelled in the legend. They are not measured trials, confidence intervals, or statistical results. All panels are conceptual, non experimental schematics representing interpretive patterns rather than data derived trajectories.

The three recurrent biomechanical phenotypes described in Section 4.1, Section 4.2 and Section 4.3 are summarized schematically in Figure 3. The figure provides a conceptual illustration of the characteristic movement patterns associated with each phenotype, rather than representing original experimental measurements or data-derived trajectories.

### 4.4. Integrating Phenotypes in Clinical Reasoning

These biomechanical phenotypes are not mutually exclusive and frequently coexist within the same individual [61]. A patient may exhibit altered scapular contribution alongside compensatory trunk strategies or demonstrate changes in axial rotation coupling only under specific task demands. Accordingly, phenotype-based interpretation should be viewed as a flexible framework that accommodates variability rather than as a rigid classification system.

Importantly, these phenotypes describe observed movement patterns rather than their underlying causes. Altered scapular or trunk kinematics may reflect local shoulder impairment, pain-related protective behaviour, or task-dependent adaptations, and these mechanisms may coexist. Therefore, kinematic findings alone cannot establish whether a movement alteration is a cause or a consequence of shoulder dysfunction [4,52].

For clinicians interpreting shoulder biomechanics, the value of this approach lies in its ability to contextualize quantitative outputs within functional behaviours. Rather than asking whether a specific variable is “normal” or “abnormal”, phenotype-based reasoning encourages consideration of how motion is achieved and why particular strategies emerge. This shift may support more informed clinical interpretation while acknowledging the complexity and adaptability of human movement. For clinical reference, Table 2 summarizes the key kinematic features, potential clinical relevance, and principal methodological caveats associated with each phenotype.

## 5. Same Data, Different Results

One of the principal challenges in interpreting shoulder biomechanics is that apparently similar datasets may yield divergent interpretations due to differences in modelling assumptions, tracking methods, and data processing choices. This variability does not necessarily reflect inconsistency in shoulder function, but rather differences in how motion is captured, modelled, processed, and reported. For clinicians and researchers alike, failure to recognize these sources of variability risks overinterpreting isolated findings or drawing conclusions that are not transferable across contexts [5].

**Figure 4:**
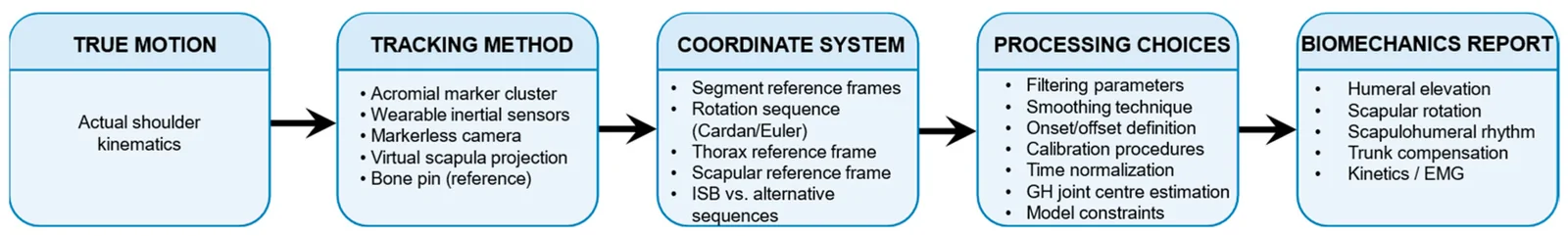
Schematic pathway illustrating how the same underlying shoulder motion may yield different reported biomechanical outputs depending on methodological choices. Actual shoulder kinematics are first captured through a chosen tracking method, then decomposed according to coordinate system and rotation conventions, further shaped by processing choices such as filtering, calibration, onset definition, normalization, and joint-centre estimation, and finally expressed as reported biomechanical variables. The figure emphasizes that outputs such as humeral elevation, scapular rotation, scapulohumeral rhythm, trunk compensation, and kinetics or sEMG reflect method-dependent representations rather than direct readouts of true motion.

In contrast to lower-limb gait analysis, where methodological conventions are relatively mature, shoulder biomechanics remains particularly sensitive to analytical choices. This sensitivity is amplified by the anatomical complexity of the shoulder complex, the absence of a fixed base of support, and the reliance on indirect methods to track scapular motion. Consistent with this variability, meta-analytic synthesis indicates that reported scapular kinematic alterations in shoulder impingement do not converge toward a single reproducible pattern, but vary according to task plane, elevation range, population characteristics, and methodological choices [62]. As a result, identical biomechanical constructs, such as scapular upward rotation or scapulohumeral rhythm, may represent subtly different phenomena depending on how they are derived. This derivation pathway and its major sources of interpretive variability are summarized schematically in Figure 4.

### 5.1. Scapular Tracking Methods and Their Interpretive Consequences

Scapular kinematics represent one of the most informative yet methodologically vulnerable domains in shoulder biomechanics. Because the scapula is covered by substantial soft tissue and moves relative to both the thorax and the humerus, its motion cannot be tracked directly using standard skin-mounted markers without approximation [47,63,64].

Different scapular tracking approaches introduce distinct, non-random error profiles that increase with movement amplitude and elevation angle [64]. Methods based on acromial marker clusters allow dynamic tracking throughout arm elevation but are increasingly sensitive to soft-tissue artefact at higher elevation angles [26,64,65]. In contrast, static or quasi-static calibration approaches provide improved anatomical accuracy at specific postures but cannot fully capture continuous motion during dynamic tasks. Variations in calibration procedures applied to acromial marker clusters have been shown to produce meaningfully different scapular kinematic outputs within the same individual, indicating that tracking-related variability persists even under controlled experimental conditions [66]. Virtual scapula projection approaches have been shown to provide more anatomically sensitive estimates of ST motion than standard acromial marker configurations [67]. Wearable inertial measurement units (IMUs) and magneto-inertial measurement units (MIMUs) offer practical advantages for portable and field-based assessments but introduce additional measurement challenges. Studies indicate that sensor placement on the scapula or upper arm represents an additional source of variability, with small differences in positioning propagating into measurable changes in scapular kinematics [68]. Additional limitations include drift, calibration errors, and misalignment with anatomical reference frames [27,69]. In MIMUs, nearby ferromagnetic objects and electromagnetic disturbances can distort magnetometer measurements, resulting in inaccurate heading estimates. Gyroscope bias and integration errors may also accumulate over time, particularly during prolonged recordings when reliable heading correction is unavailable. Sensor-to-segment misalignment represents another important source of measurement uncertainty because sensor orientation does not necessarily coincide with the anatomical axes of the underlying body segment. To improve accuracy and reproducibility, calibration protocols should establish the relationship between sensor and anatomical coordinate systems using standardized postures, functional movements, or anatomical landmark identification, as appropriate for the clinical population. Furthermore, researchers should document sensor placement, calibration procedures, magnetic environment, recording duration, and drift-correction methods when reporting shoulder kinematics [27,68,69]. Beyond wearable inertial systems, consumer-grade and immersive motion-tracking technologies have recently been explored for shoulder kinematic assessment, demonstrating promising feasibility but accuracy limitations that remain dependent on task execution and experimental setup [70].

Quantitative findings further illustrate the methodological variability of scapular tracking. For acromial marker clusters, a systematic review reported average errors of approximately 5° during arm flexion and 7° during abduction below 90–100° of humeral elevation, with additional calibration recommended at higher angles [64]. For wearable magneto-inertial sensors, reported accuracy varies according to sensor placement and movement conditions. For example, a root mean square error (RMSE) of 25.6° was reported for scapular internal–external rotation during an overhead lifting task when compared with optical motion capture [27]. Regarding virtual scapula projection methods, Janes et al. reported mean scapular upward rotation ranges of 57° for virtual projection and 37° for surface markers during scaption, representing a statistically significant difference between methods (*p* < 0.0055) rather than a measurement error relative to an anatomical reference standard [67]. Overall, these findings highlight the dependence of scapular kinematic estimates on the tracking method, calibration procedures, and movement conditions, particularly at higher elevation angles, and the difficulty of directly comparing accuracy across technologies using different validation methods.

From an interpretive perspective, these differences mean that scapular kinematic outputs should be understood as method-dependent representations rather than absolute measures [28]. Apparent discrepancies between studies may therefore reflect differences in tracking fidelity or elevation range rather than true biomechanical divergence [63]. Recognizing this limitation is essential when comparing scapular motion patterns across tasks, populations, or experimental setups.

### 5.2. Coordinate Systems, Rotation Sequences, and Reference Frames

Beyond tracking methods, the mathematical conventions used to describe shoulder motion can substantially influence reported kinematic outcomes. Choices related to coordinate system definition, segment reference frames, and rotation sequence (e.g., Cardan or Euler angles) influence how three-dimensional motion is decomposed into clinically interpretable components [6,8].

These choices are particularly relevant at the shoulder, where large three-dimensional rotations occur throughout many functional tasks. Differences in rotation sequence or axis definition can alter how the same underlying motion is represented across individual rotational components, affecting the magnitude and temporal profile of reported variables [64]. This effect has been demonstrated across a wide range of tasks and experimental contexts. The choice of rotation sequence alone has been shown to produce substantially different shoulder kinematic curves from identical underlying motion data, an effect that is particularly pronounced during high-velocity overhead tasks where gimbal lock becomes a practical risk [71]. More recent work confirms that, although rotation sequences recommended by the International Society of Biomechanics reduce mathematical singularities, alternative sequences remain widely used and can transform identical underlying motion into divergent kinematic representations, resulting in variables that are not directly comparable across studies [72]. Importantly, rotation sequence effects may compound with other methodological choices, such that differences in both tracking method and rotation convention jointly alter reported HT kinematics even when the same motion capture system is used [73]. Consequently, two studies reporting the same named variable may be describing different underlying motions [63].

More specifically, the ISB recommendations propose a Y–X′–Y″ Euler rotation sequence for describing humerothoracic and glenohumeral orientation [6]. The three successive rotations represent the plane of elevation, the elevation angle, and humeral axial rotation, respectively. However, this representation is susceptible to gimbal lock when the middle rotation approaches 0° or 180°, because the first and third rotational axes become aligned, making their angular contributions difficult to distinguish. Alternative Cardan sequences, such as X–Z′–Y″, may reduce the occurrence of gimbal lock in specific tasks, as demonstrated during tennis serves [71]. However, other investigations have found the ISB-recommended Y–X′–Y″ sequence to be more appropriate for volleyball attacks [72]. Furthermore, different rotation sequences can produce substantially different angular trajectories from the same underlying motion [73]. Therefore, the selection of a rotation sequence should consider the specific movement task, susceptibility to mathematical singularities, and anatomical interpretability. To support reproducibility and cross-study comparisons, researchers should explicitly report the anatomical coordinate systems, rotation sequence, axis conventions, and procedures used to identify and manage angular discontinuities [6,63,71,72,73].

For clinical interpretation, biomechanical variables should therefore be considered in relation to the conventions used to derive them rather than treated as interchangeable measures. Understanding how a variable was defined is often as important as knowing what was measured, particularly when comparing kinematic findings across studies [6,11].

### 5.3. Data Processing and Signal Treatment

Data processing choices represent another major source of variability in shoulder biomechanics. Filtering parameters, smoothing techniques, and time normalization procedures can materially alter the appearance of kinematic curves, the detection of peak events, and the estimation of coordination metrics [74]. Common processing approaches include low-pass filtering of kinematic trajectories to reduce high-frequency noise, with filter type, order, and cut-off frequency selected according to the measurement system, sampling frequency, and movement characteristics. For reproducibility, studies should report these parameters, together with the application of zero-phase filtering when appropriate, as inappropriate filtering choices may alter peak amplitudes, movement timing, and intersegmental coordination estimates [74]. Glenohumeral joint centre estimation may rely on predictive or functional methods, with potential estimation errors influencing the accuracy of subsequent kinematic and kinetic calculations. Errors in anatomical parameter estimation, such as mislocation of the GH joint centre by as little as 5–10 mm, have been shown to propagate substantially into both joint angle and joint moment calculations, amplifying small identification errors into meaningful kinematic and kinetic differences [75].

Additional considerations apply to wearable inertial systems. Calibration procedures and model constraints may influence the estimation of segment orientation and joint kinematics [76], while measurement accuracy may also vary according to the task performed and the modelling assumptions adopted [77]. Validation studies further indicate that measurement error can depend on the range of motion examined, warranting caution when comparing absolute kinematic values across measurement approaches [78]. Temporal normalization introduces a further source of variability. The definition of movement onset, peak elevation, and task completion can influence the temporal alignment of biomechanical curves across trials and consequently affect the interpretation of coordination patterns [74]. These considerations become particularly relevant when derived metrics, such as scapulohumeral rhythm, depend on multiple processed kinematic signals.

From an interpretive perspective, numerical values and temporal patterns should therefore be considered in relation to the processing, calibration, and modelling procedures from which they were derived. Methodological transparency is essential when comparing biomechanical outputs across studies or measurement systems.

### 5.4. Implications for Clinical and Research Interpretation

The combined effects of tracking methods, modelling conventions, and processing choices mean that the same underlying movement may yield different reported biomechanical outputs. This variability does not undermine the value of biomechanical assessment; rather, it underscores the importance of interpreting biomechanical outputs in relation to the methodological choices through which they were derived [5,8,64].

For clinicians, biomechanical reports should be interpreted by integrating individual outputs with task context, intersegmental coordination, and compensatory movement strategies rather than considering isolated metrics [3,4,79]. For researchers, this variability highlights the need for methodological transparency and caution when generalizing findings across studies or proposing normative benchmarks [6].

Acknowledging methodological variability is therefore essential for the meaningful interpretation and translation of biomechanical findings. Explicit consideration of the factors that shape biomechanical outputs can support a more informed functional interpretation of shoulder movement without overstating the precision or comparability of individual measures. The main methodological factors affecting shoulder biomechanical measurements and their implications for interpretation are summarized in Table 3.

### 5.5. Emerging Markerless and Camera-Based Systems

Recent advances in markerless and camera-based motion capture systems have expanded the accessibility of shoulder kinematic assessment beyond specialized laboratories. Validation studies demonstrate that markerless approaches can provide acceptable accuracy for overall humeral kinematics during functional upper-limb tasks, although performance remains dependent on task characteristics and experimental setup [13,14]. Importantly, camera geometry has emerged as a critical methodological factor, with viewing angle and camera placement substantially influencing kinematic accuracy, particularly for scapular motion, where posterior and oblique perspectives yield lower error than frontal views [15]. While these technologies offer practical advantages for clinical and field-based assessment, current evidence indicates that they introduce new sources of variability related to camera configuration and environmental constraints and should therefore be interpreted cautiously when applied to fine-grained ST analysis.

An important distinction should be made between multi-camera markerless motion capture systems and single-view RGB-D configurations. Multi-camera systems use synchronized observations from different viewpoints to reconstruct three-dimensional movement and may reduce the effects of occlusion through complementary camera perspectives. However, their implementation requires appropriate camera calibration, synchronization, sufficient spatial resolution, and adequate coverage of the movement volume. In contrast, single-view RGB-D systems combine colour images with depth information, offering a more portable and potentially less resource-intensive solution, but their accuracy may be limited by self-occlusion, viewing angle, depth-sensor resolution, and pose-estimation uncertainty. These limitations are particularly relevant for scapular kinematics because the scapula is not directly visible and its motion is difficult to infer reliably from external body landmarks. Consequently, acceptable accuracy for overall upper-limb or humerothoracic movement should not be interpreted as evidence of equivalent accuracy for glenohumeral or scapulothoracic kinematics without independent validation [13,14,15,17].

## 6. Practical Guide: How to Read a Shoulder Biomechanics Report

Instrumented shoulder biomechanics reports often present complex datasets that can be difficult to integrate into clinical reasoning. A structured approach to interpretation is therefore essential to avoid overemphasizing isolated metrics or misattributing compensatory strategies to primary shoulder dysfunction. The following stepwise framework is intended to guide clinicians through the interpretation of shoulder biomechanics reports in a consistent and clinically meaningful manner.

**Figure 5:**
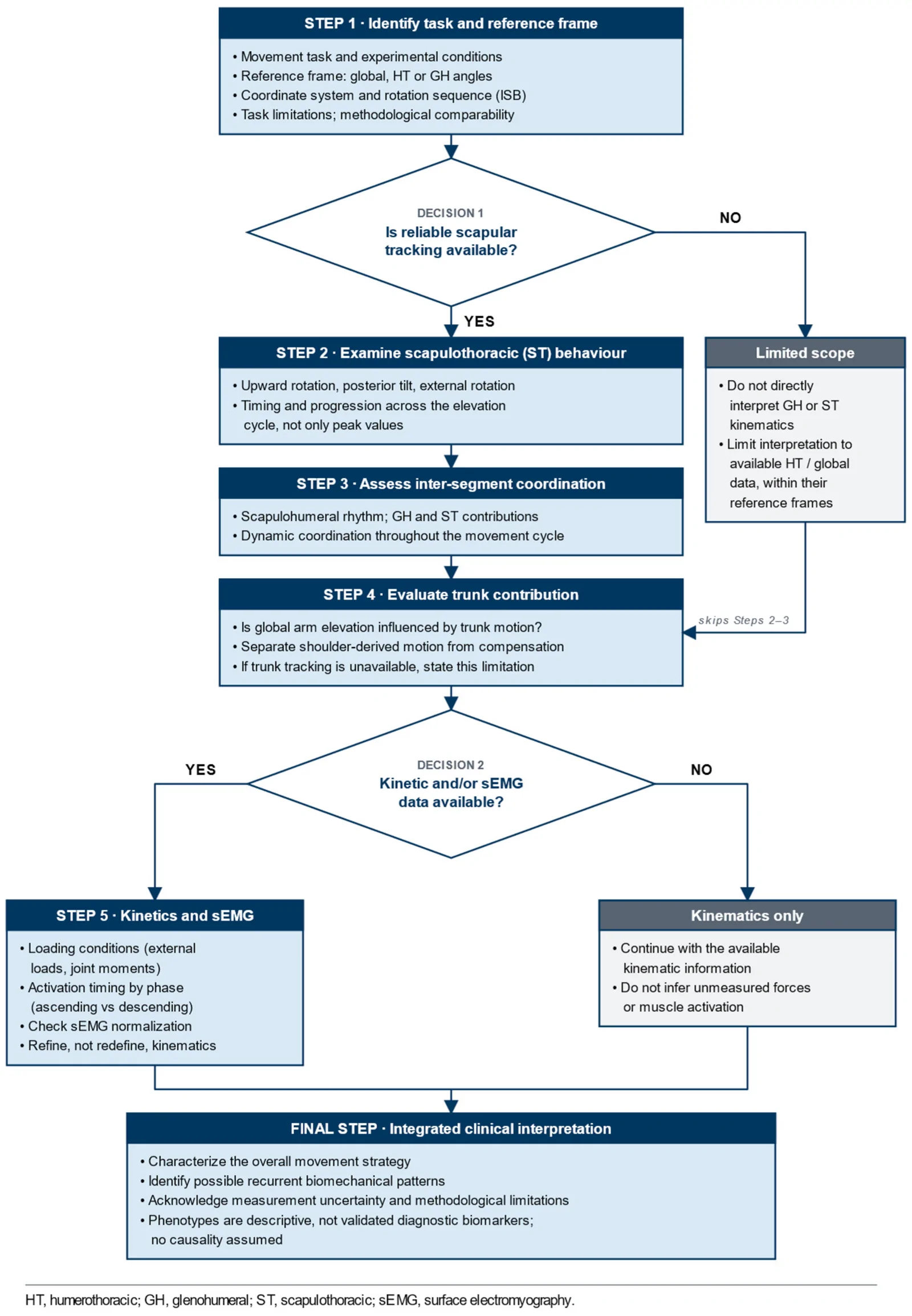
Conceptual clinical decision flowchart for the interpretation of shoulder biomechanics reports. The pathway summarizes the five-step framework presented in Section 6, beginning with identification of the movement task and reference frame. Depending on the availability of reliable scapular tracking, the assessment proceeds through scapulothoracic kinematics and inter-segment coordination or is restricted to the available humerothoracic and global measures. Trunk contribution is subsequently evaluated, followed by integration of kinetic and surface electromyographic data when available. The final step combines the available biomechanical information into a task-dependent functional interpretation while acknowledging measurement uncertainty and methodological limitations. The flowchart represents a conceptual interpretive aid rather than a validated diagnostic algorithm.

The proposed clinical interpretation pathway is summarized in Figure 5. The flowchart guides readers through task identification, verification of available segmental measurements, assessment of scapular and humeral coordination, evaluation of trunk compensation, and integration of kinetic and neuromuscular information when available. The pathway is intended as a conceptual decision aid rather than a validated diagnostic algorithm.

### 6.1. Step 1: Identify the Task and Its Reference Frame

Interpretation should begin by clearly identifying the task performed and the reference frame used for reporting motion. Scapular-plane elevation can be interpreted within a standardized elevation cycle, whereas functional reaching, lifting, or resisted tasks impose additional mechanical and coordinative demands. Without this contextual information, comparisons across reports or time points are unreliable [6].

Clinicians should verify whether reported angles describe motion relative to the trunk, the scapula, or a global reference frame, as this distinction fundamentally alters the meaning of apparent ROM and coordination.

### 6.2. Step 2: Examine Scapulothoracic Behaviour Before Humeral Motion

ST kinematics should be assessed before interpreting humeral or GH motion. Because the scapula provides the mobile base for arm elevation, alterations in scapular contribution can profoundly influence apparent humeral kinematics [24].

Rather than focusing on peak angles, clinicians should examine the timing and progression of scapular upward rotation, posterior tilt, and external rotation across the elevation cycle. Delayed or redistributed scapular contribution may signal altered control strategies even when terminal values appear unremarkable.

### 6.3. Step 3: Assess Inter-Segment Coordination

Once scapular and humeral motions have been reviewed individually, attention should shift to their coordination. Scapulohumeral rhythm provides insight into how motion is shared between segments and often reveals functional adaptations that are not evident from isolated curves.

Coordination should be interpreted dynamically, with particular attention to changes across different phases of the task. Fixed ratios or single summary values should be interpreted cautiously, as they may obscure meaningful temporal variations in coordination.

### 6.4. Step 4: Consider Trunk Contribution and Compensation

Apparent preservation of shoulder ROM may be achieved through compensatory trunk movements. Clinicians should therefore verify whether thoracic motion has been quantified and whether humeral elevation is referenced to the trunk or to a global frame [52].

Failure to account for trunk contribution may lead to overestimation of true shoulder function and under recognition of compensatory strategies that influence symptom persistence or rehabilitation outcomes.

A practical approach to distinguishing shoulder-related limitations from compensatory trunk contributions involves examining segmental motion during the same task. When both global and segment-referenced kinematics are available, clinicians should compare global arm elevation with humerothoracic motion and assess concurrent trunk displacement. Preserved global arm elevation accompanied by reduced humerothoracic excursion and increased trunk movement may indicate that trunk motion contributes to task completion despite limited shoulder-relative mobility [52,53]. When reliable scapular tracking is available, examination of GH and ST contributions may further characterize how motion is distributed within the shoulder complex. Repeated assessment under comparable task conditions, including trials with reduced trunk movement when feasible, may provide additional information on the flexibility of the observed strategy. However, changes induced by trunk restriction should not be interpreted as a validated diagnostic test or as evidence of causality. Kinematic findings must be considered alongside symptoms, active and passive range of motion, strength, and the broader clinical examination to determine whether the observed pattern is consistent with an underlying shoulder impairment or a context-dependent adaptation [4,52].

### 6.5. Step 5: Integrate Kinetic and Neuromuscular Information When Available

When reports include kinetic data or electromyographic measures, these should be used to refine, rather than redefine, interpretation. External loads and joint moments provide context regarding mechanical demand, while sEMG timing patterns may help explain observed kinematic strategies.

Given their methodological sensitivity, these variables should not be interpreted in isolation. Their primary value lies in supporting or clarifying kinematic and coordination-based observations.

### 6.6. Common Interpretive Pitfalls

Several recurrent pitfalls should be avoided when interpreting shoulder biomechanics reports, including treating isolated kinematic variables as diagnostic indicators, comparing absolute values across studies without considering methodological differences, overlooking compensatory trunk strategies, and overinterpreting sEMG amplitude without normalization context. Awareness of these limitations helps ensure that biomechanical data inform clinical reasoning without overstating precision or certainty.

## 7. Study Limitations and Future Directions

### 7.1. Conceptual and Methodological Limitations

The interpretive framework proposed in this review is constrained by the methodological heterogeneity of current shoulder biomechanics research. Differences in task execution, scapular tracking, coordinate system conventions, and data processing limit direct comparisons across studies and laboratories [8,80].

The assessment of scapular motion represents a particular methodological challenge. Non-invasive tracking approaches are affected by soft-tissue artefact and may also be influenced by elevation angle and calibration procedures [26]. Scapulothoracic kinematic outputs should therefore be interpreted in relation to the measurement approach used, particularly when comparing absolute values across studies or considering normative thresholds.

A further limitation arises from the complexity of shoulder function within the broader kinetic chain. During functional and high-demand tasks, shoulder movement may interact with trunk and lower-limb contributions, and focusing exclusively on local shoulder kinematics may overlook relevant compensatory or whole-body movement strategies [52].

Finally, this review adopts a narrative rather than a systematic approach and does not provide exhaustive coverage of the literature or quantitative estimates of effect magnitude. The proposed framework should therefore be regarded as an interpretive approach rather than as a prescriptive or validated classification.

### 7.2. Directions for Methodological Standardization

Future progress in shoulder biomechanics will require greater consistency and transparency in methodological reporting. Clear documentation of task execution, coordinate systems, rotation sequences, tracking methods, and data processing choices would facilitate comparison across studies and improve reproducibility [6].

Consensus-driven reporting guidelines may further support the interpretation of shoulder biomechanical data. Such guidelines should promote methodological transparency and a shared descriptive language without requiring uniform measurement approaches, recognizing that different research and clinical contexts may require different assessment solutions.

### 7.3. Advancing Clinical Translation

Translating biomechanical assessment into clinically meaningful information remains an important challenge. Future research should investigate whether recurrent biomechanical phenotypes are associated with clinically relevant outcomes, including symptom trajectories, functional recovery, and responses to intervention. Longitudinal studies will be particularly important to determine whether these patterns change alongside clinical status and whether they provide information beyond conventional clinical measures.

Clinical translation also requires accessible approaches to interpreting increasingly complex biomechanical outputs. Frameworks that emphasize movement coordination, compensatory strategies, and task context may facilitate the integration of biomechanical information with clinical assessment without reducing shoulder function to isolated metrics.

### 7.4. Emerging Technologies and Opportunities

Advances in wearable sensors, markerless motion capture, and data-driven modelling may expand shoulder biomechanical assessment beyond specialized laboratory environments [81,82]. These technologies offer opportunities for movement assessment in more naturalistic settings and over longer periods, although their application introduces additional methodological and interpretive challenges [27].

For clinical implementation, the validation of wearable and markerless technologies should extend beyond overall measurement accuracy to include task-specific reliability, sensitivity to sensor placement and camera configuration, and the stability of measurements during prolonged or repeated assessments. In particular, the ability to estimate humerothoracic motion should be distinguished from the more demanding assessment of glenohumeral and scapulothoracic kinematics, which requires reliable information on scapular orientation. Standardized calibration, transparent reporting of measurement uncertainty, and validation against appropriate reference methods are therefore essential before these technologies can support reproducible clinical interpretation [12,16,17,27].

Emerging analytical approaches, including coordination metrics and machine learning-based pattern recognition, may further support the characterization of multidimensional biomechanical data. Future development should prioritize transparent and interpretable approaches that complement, rather than replace, biomechanical and clinical reasoning.

## 8. Conclusions

The value of instrumented shoulder biomechanical assessment lies not in the number of variables reported, but in their integrated interpretation within the context of the movement task. Kinematic, coordination, kinetic, and neuromuscular outputs provide complementary information that should be considered together to characterize how shoulder movement is achieved.

This narrative review proposes an interpretive framework organized around a reference elevation task, complementary biomechanical domains, and recurrent functional phenotypes. The shoulder elevation cycle provides a common temporal scaffold for interpretation without prescribing how biomechanical data should be collected. By emphasizing movement coordination, compensatory strategies, and task context rather than isolated metrics or rigid thresholds, the proposed framework supports a multidimensional interpretation of shoulder function while acknowledging the methodological variability inherent to current measurement approaches.

For rehabilitation clinicians, three practical considerations follow from this framework. First, biomechanical outputs should be interpreted in relation to the task, reference frame, and measurement method rather than as isolated indicators of normality or pathology. Second, scapular, humeral, and trunk contributions should be considered together to distinguish local shoulder motion from compensatory movement strategies. Third, recurrent biomechanical phenotypes may be used as descriptive movement strategies to contextualize and monitor shoulder function, rather than as diagnostic biomarkers or causal explanations of symptoms. Their prognostic and treatment-related value remains to be established through longitudinal research.

## Figures and Tables

**Table 1:** Database-specific literature search strategies.

Database	Search Strategy
**PubMed**	((“Shoulder Joint”[MeSH] OR “Scapula”[MeSH] OR shoulder[Title/Abstract] OR glenohumeral[Title/Abstract] OR scapulothoracic[Title/Abstract] OR scapula[Title/Abstract]) AND (“Biomechanical Phenomena”[MeSH] OR “Kinematics”[MeSH] OR “Kinetics”[MeSH] OR biomechanics[Title/Abstract] OR kinematic*[Title/Abstract] OR kinetic*[Title/Abstract] OR “motion analysis”[Title/Abstract] OR “movement analysis”[Title/Abstract]) AND (“motion capture”[Title/Abstract] OR optoelectronic[Title/Abstract] OR electromyography[Title/Abstract] OR EMG[Title/Abstract] OR “inertial measurement unit”[Title/Abstract] OR IMU[Title/Abstract] OR dynamometry[Title/Abstract]) AND (marker set[Title/Abstract] OR inverse dynamics[Title/Abstract] OR joint angle*[Title/Abstract] OR biomechanical model*[Title/Abstract] OR segment*[Title/Abstract] OR coordinate system*[Title/Abstract]))
**Scopus**	(shoulder OR glenohumeral OR scapulothoracic OR scapula) AND (biomechanics OR kinematics OR kinetics OR “motion analysis” OR “movement analysis”) AND (“motion capture” OR optoelectronic OR electromyography OR EMG OR “inertial measurement unit” OR IMU OR dynamometry) AND (“marker set” OR “inverse dynamics” OR “joint angle*” OR “biomechanical model*” OR segment* OR “coordinate system*”)
**Web of** **Science**	TS = ((shoulder OR glenohumeral OR scapulothoracic OR scapula) AND (biomechanics OR kinematics OR kinetics OR “motion analysis” OR “movement analysis”) AND (“motion capture” OR optoelectronic OR electromyography OR EMG OR “inertial measurement unit” OR IMU OR dynamometry) AND (“marker set” OR “inverse dynamics” OR “joint angle*” OR “biomechanical model*” OR segment* OR “coordinate system*”))

**Table 2:** These phenotypes represent recurrent interpretive patterns rather than formally derived clusters or validated diagnostic classifications. They may coexist within the same individual, and their interpretation should account for task demands and methodological variability.

Functional Phenotype	Key Kinematic Signatures	Potential Clinical Manifestations and Relevance	Major Methodological Caveats
**Altered scapular contribution**	Altered timing and magnitude of scapulothoracic motion relative to humeral elevation; changes in scapular upward rotation and posterior tilt; reduced early scapular contribution potentially followed by compensatory increases at higher elevation angles; changes may occur despite similar peak scapular angles.	May be associated with shoulder pain or altered neuromuscular function; may reflect task-dependent or adaptive movement strategies rather than pathology; relevant to assessing dynamic scapular control and guiding rehabilitation focused on movement timing and coordination.	Scapular tracking is affected by soft-tissue artefact, tracking technique, calibration, and elevation angle; peak angles alone may overlook changes in movement timing and progression; findings should be interpreted in relation to the task and measurement method.
**Compensatory trunk–shoulder strategy**	Increased trunk extension, lateral flexion, or axial rotation during arm elevation; preserved or exaggerated humeral elevation in the global reference frame; reduced or delayed glenohumeral and scapulothoracic contributions relative to trunk motion; redistribution of movement across the trunk and upper limb.	May enable task completion despite reduced shoulder contribution; can mask underlying shoulder limitations and lead to overestimation of functional range of motion; relevant to distinguishing true shoulder function from compensatory whole-body movement strategies.	Interpretation depends on the reference frame used to calculate humeral elevation; without concurrent trunk-motion assessment, compensation may be mistaken for preserved shoulder function; segmental contributions must be distinguished from overall task performance; findings are task-dependent and not diagnosis-specific.
**Altered axial rotation and coupling**	Reduced variability in humeral axial rotation; delayed rotational adjustments during elevation; altered temporal coupling between elevation and axial rotation; changes in rotational timing or coordination despite relatively preserved peak angular values.	May be associated with shoulder pain, fatigue, or high-demand functional tasks; may reveal altered movement control not captured by peak range-of-motion measurements; provides complementary information for assessing dynamic coordination, although its mechanical and clinical implications remain incompletely established.	Static measurements and peak angular values may fail to capture temporal coupling; interpretation depends on task demands and measurement conventions; coordination measures should be assessed alongside scapular and humeral kinematics; mechanical implications and relationships with clinical outcomes remain incompletely established.

**Table 3:** Main methodological factors affecting shoulder biomechanical measurements and their implications for interpretation.

Methodological Factor	Potential Effect on Measurements	Practical Implication for Interpretation
Scapular tracking method and soft-tissue artefact	Tracking approaches have different error profiles; errors may increase with movement amplitude and elevation angle. Calibration choices and sensor placement can also change estimated scapular kinematics.	Treat scapular kinematic outputs as method-dependent representations, not absolute measures. Apparent differences between studies may reflect tracking fidelity, elevation range, calibration, or sensor placement rather than true biomechanical differences.
Coordinate systems, segment reference frames, and rotation sequences	Different axis definitions or Cardan/Euler rotation sequences can change the magnitude and temporal profile of reported rotational components, even when the underlying motion is identical.	Do not assume that identically named variables are directly comparable across studies. Interpret each variable in relation to the conventions used to derive it.
Filtering, smoothing, and temporal normalization	Processing choices can alter kinematic curve profiles, peak-event detection, and coordination metrics. Definitions of movement onset, peak elevation, and task completion affect temporal alignment.	Interpret curve shape, peak timing, and coordination patterns in the context of the processing and event definitions used.
Calibration, anatomical parameter estimation, and model assumptions	Calibration procedures and model constraints can affect segment-orientation and joint-kinematic estimates. Errors in anatomical parameter estimation can propagate into joint-angle and joint-moment calculations.	Consider calibration and modelling assumptions when interpreting kinematic and kinetic outputs, and avoid treating derived values as independent of the methods used to estimate them.
Measurement system and task context	Wearable inertial, consumer-grade, and immersive tracking approaches have accuracy limitations that may depend on task execution and experimental setup; wearable systems may also be affected by drift and alignment with anatomical reference frames.	Account for the measurement approach and task conditions when comparing results across systems or generalizing findings to other tasks, populations, or settings.

**Note.** The table synthesizes methodological effects described in Section 5.1, Section 5.2, Section 5.3, Section 5.4 and Section 7.1. It is intended as a qualitative summary and does not introduce new quantitative estimates or imply fixed errors across methods.

## Data Availability

No new data were created or analyzed in this study. Data sharing is not applicable to this article.
